# LGBTQ PROGRAMMING AT SENIOR CENTERS IN MASSACHUSETTS

**DOI:** 10.1093/geroni/igz038.563

**Published:** 2019-11-08

**Authors:** Ceara Somerville, Saralyn Collins, Caitlin E Coyle

**Affiliations:** 1 University of Massachusetts Boston, Boston, Massachusetts, United States; 2 Gerontology Institute, University of Massachusetts Boston, Boston, Massachusetts, United States

## Abstract

LGBTQ seniors have some different needs for programs and services, are at a higher risk of social isolation, and are often underserved in the community. Senior centers serve as a hub of resources in a community and are purposefully situated to address the needs and interests of all seniors in a community; they are a natural outlet for targeted programming for LGBTQ seniors. The purpose of this project is to demonstrate what municipal senior centers across Massachusetts are doing to meet the needs of their LGBTQ seniors. A total of 24 senior centers were identified by the Massachusetts Association of Councils on Aging (MCOA) as providing LGBTQ programming. Semi-structured interviews were conducted with 14 senior center directors or programming staff from different communities across Massachusetts to learn more about their specific programming. For almost all senior centers in this study, the main LGBTQ-specific programming was a congregate meal with an activity. Activities included both recreational activities like a film-screening and educational engagements such as guest speakers or specialists on housing, legal services, and health promotion. Distinguishing characteristics included whether or not programming had an intergenerational component, type of recruitment methods, and geographic clustering of programs. For example, two regions emerged as having shared activities for LGBTQ seniors. Results from this study will be used to illustrate models of best practice when it comes to LGBTQ programming for older people.

